# Feminizing adrenocortical adenoma in a girl from a resource-limited setting: a case report

**DOI:** 10.1186/s13256-021-03203-8

**Published:** 2021-12-21

**Authors:** Eman Abdalla Ali Elnaw, Areej Ahmed Bashier Ibrahim, Mohamed Ahmed Abdullah

**Affiliations:** 1 The Endocrine Division, Department of Pediatrics and Child Health, Faculty of Medicine, University of Khartoum, P.O. Box 102, Khartoum, Sudan; 2 Jafar Ibn Auf Pediatric Tertiary Hospital, Khartoum, Sudan

**Keywords:** Feminizing adrenocortical tumor, Precocious puberty, Resource-limited setting, Case report

## Abstract

**Background:**

An adrenocortical tumor is a rare tumor in pediatrics, which can be functional or nonfunctional. Functional tumors present with virilization, feminization, or hypercortisolism. Feminizing adrenal tumors, though rare in pediatrics, need to be excluded in any child presenting with features of feminization.

**Case presentation:**

We report a case of a 4-year-old Sudanese girl who presented with gradually progressive bilateral breast enlargement and accelerated growth since the age of 6 months. The family had sought medical advice several times in numerous health facilities without much gain. Investigations showed pubertal luteinizing hormone levels, high estradiol E2, and dehydroepiandrosterone sulfate, with normal early morning cortisol level. Abdominal ultrasound revealed a right-sided hypoechoic suprarenal mass. Abdominal computed tomography scan showed a right adrenal mass. The diagnosis of feminizing adrenal neoplasm was confirmed and right adrenalectomy was done. Histopathological examination of the resected adrenal gland showed adrenocortical adenoma. The patient was started on gonadotrophin-releasing hormone agonist for secondary central precocious puberty.

**Conclusion:**

Adrenocortical tumors, though rare in pediatrics, are a documented cause of precocious puberty; biochemical and imaging screening protocol should be adopted for patients with precocious puberty, even in a resource-limited setting, for early detection and treatment.

## Background

An adrenocortical tumor (ACT) is a rare disease in pediatrics. The annual worldwide incidence is 0.3–0.38 per million children, with a peak incidence in those younger than 5 years [1].

ACT can be functional or nonfunctional. Functional tumors can present with peripheral precocious puberty with predominant virilization or feminization, hypercortisolism alone or in combination with peripheral precocious puberty, or rarely with hypoaldosteronism [1].

Owing to the rarity of these tumors and their variable presentation, they can be missed by general physicians and pediatricians in primary care facilities, and this is what had happened to our patient. She had suffered from progressive peripheral isosexual precocious puberty caused by long-lasting excess estrogen production from a right adrenal tumor, which had been complicated by central precocious puberty as a result of the delayed diagnosis and management.

We report this case to show the importance of adopting biochemical and imaging screening for early detection of ACT, and the need to train doctors in primary health care facilities in our limited-resource setting about the causes of precocious puberty and the importance of early diagnosis and management.

## Case presentation

A 53-month-old Sudanese female presented with progressive bilateral breast enlargement and accelerated growth since the age of 9 months. Her family had sought medical advice several times in different primary health care facilities and were reassured. She had no vaginal bleeding and no pubic or axillary hair.

Examination showed a well-looking girl, vitally stable with normal blood pressure. Her weight was 17 kg (50th centile) and height 108 cm (90th centile) using the Centers for Disease Control and Prevention growth chart. Mid-parental height was 175 cm and predicted adult height was 167 cm using the JM Tanner formula. No previous documented follow-up growth data were available. Her Tanner staging was A_1_, P_1_, and B_3_. She had reddish mucoid vagina. She had no clitoromegaly, acne, hirsutism, or palpable abdominal mass (Table 1).

**Table 1:** Timeline of case presentation

Date	Relevant past medical history and intervention
Before 2020	The family noticed bilateral breast enlargement, sought medical advice in different primary care facilities and had been reassured

Date	Summary	Testing	Interventions
First visit: 8 December 2020	Patient presented at the age of 4 years and 5 months with gradually progressive bilateral breast enlargement since the age of 9 months, with accelerated growth	Left wrist X-ray: bone age of 8 years	Hormonal tests and abdominal ultrasound requested
Second visit: 15 December 2020	Investigations and imaging results	Basal luteinizing hormone 3.1 mIU/L prestimulation. Increased to 8.8 mIU/L 45 minutes post-gonadotrophin-releasing hormone stimulation Estradiol E2 29000 pg/mL (5–15 pg/mL) Dehydroepiandrosterone sulfate 90 ng/mL (2.3 ng/mL). Early morning cortisol level 16 ng/mL (7–28 ng/ml) Abdominal ultrasound showed a right-sided hypoechoic suprarenal mass	Abdominal CT scan requested.
Third visit 22 December 2020	Follow-up with abdominal CT scan result	Abdominal CT scan revealed a well-defined rounded focal lesion with a smooth outline at the level of the right adrenal gland with normal left adrenal gland and ovaries	Diagnosis of estrogen-secreting adrenocortical tumor and surgical referral
Fourth visit 12 January 2021	Postoperative evaluation, patient was clinically well	Normal cortisol and dehydroepiandrosterone sulfate. Estradiol E2 40 pg/mL consistent with central precocious puberty.	Patient started on gonadotrophin-releasing hormone agonist
Fifth visit 15 June 2021	Patient was well, compliant to monthly gonadotrophin agonist injections with partial regression of her secondary sexual characters and normal growth velocity (5 cm/year)	Abdominal CT scan was normal with no evidence of recurrence	To continue on gonadotrophin-releasing hormone agonist, with regular follow–up for the possibility of tumor recurrence

Left wrist X-ray revealed a bone age of 8 years.

The hormonal evaluation using fluorometric enzyme immunoassay showed basal luteinizing hormone of 3.1 mIU/L, which increased to 8.8 mIU/L 45 minutes post-gonadotrophin-releasing hormone stimulation. Elevated levels of estradiol E2 29,000 pg/ml (5–15 pg/ml), and dehydroepiandrosterone sulfate 90 ng/mL (2.3 ng/mL), with normal early morning cortisol level 16 ng/mL (7–28 ng/mL). Due to financial difficulties, we did not measured the follicular-stimulating hormone level.

**Fig. 1:**
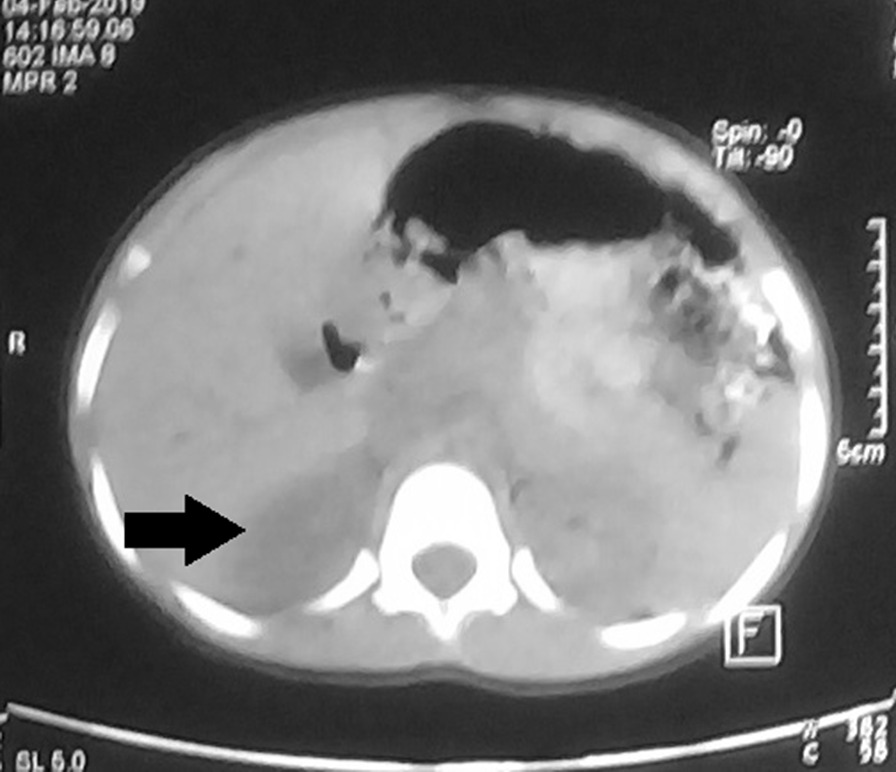
Abdominal computed tomography scan showing a right adrenal tumor (arrow)

Abdominal ultrasound revealed a right-sided hypoechoic suprarenal mass, an ovarian volume of 1.8 cm^3^, uterine volume of 3 cm^3^, and endometrial thickness of 1.2 cm. The abdominal CT scan showed a 25 × 22 mm well-defined rounded focal lesion with a smooth outline, at the level of the right adrenal gland with homogeneous attenuation, HU-7 on a noncontrast scan, and no evidence of local tissue invasion (Fig. 1). The left adrenal gland and ovaries were normal.

Brain magnetic resonance imaging was done to exclude a central cause of precocious puberty and was normal. Complete hemogram, liver, and renal functions were normal.

A diagnosis of an estrogen-secreting right adrenocortical tumor was made, and we referred the patient to surgery.

During laparotomy a 3 cm diameter tumor in the right adrenal gland was completely excised. The histopathological examination showed well-circumscribed tumor forming nests with trabeculae and sheets of polygonal cells with eosinophilic cytoplasm. The tumor was not infiltrating the capsule, with no mitosis, atypia, or necrosis (Wieneke index score = 0). Findings suggestive of benign adrenal cortical adenoma.

The postoperative evaluation revealed normal cortisol and dehydroepiandrosterone sulfate. Estradiol E2 40 pg/mL was consistent with secondary central precocious puberty, so the patient was started on monthly gonadotrophin-releasing hormone agonist, with a regular follow-up plan for the possibility of recurrence of adrenal adenoma.

Six months after the operation, the patient was well and compliant to monthly gonadotrophin agonist injections. She showed partial regression of her secondary sexual characters and a growth velocity of 5 cm/year. The abdominal CT scan was normal with no evidence of recurrence.

## Discussion

ACT is a rare disease in pediatrics. The annual worldwide incidence is 0.3–0.38 per million children, with a peak incidence in those younger than 5 years [1].

ACT can be functional or nonfunctional. Functional tumors can present with peripheral precocious puberty with predominant virilization or feminization, hypercortisolism alone or in combination with peripheral precocious puberty, or rarely it can present with hyperaldosteronism [1].

Feminizing adrenocortical tumors (FAT) are rare in pediatrics. They present mainly with peripheral precocious puberty; isosexual in girls and heterosexual in boys. Excessive estrogen secretion, with or without other adrenal hormones, is the main biochemical finding. Imaging and biochemical assessment of other adrenal hormones can discriminate adrenal from ovarian secretion of estrogen [2]. Due to the variability in the clinical presentation of these tumors and their rarity, they can be missed by doctors in primary care facilities and this is what had happened to our patient.

The etiology of ACTs is currently not very clear. Mutations in the *P53* gene are the most common inherited genetic abnormalities associated with increased ACTs frequency in familial cancer syndromes. Overexpression of steroidogenic factor-1 and insulin-like growth factor-2 plays a pivotal role in ACTs pathogenesis [3]. As we have no facility for genetic testing in Sudan, we had not tested our patient for these known genetic associations.

Li Fraumeni, Beckwith–Wiedeman syndromes, isolated hemihypertrophy syndromes, congenital anomalies of the kidney, and congenital adrenal hyperplasia are commonly associated with ACTs [3]. Our patient had no clinical evidence of any of these associations.

The laboratory characteristics of ACTs include disturbance of the normal circadian rhythm of cortisol secretion, and discordantly elevated serum levels of sexual corticosteroids including dehydroepiandrosterone sulfate, estrogen, and testosterone. Elevated testosterone is the predominant hormonal abnormality in virilizing adrenal tumors, while raised estrogen is the main biochemical abnormality in feminizing tumors [1]. Our patient presented with clinical features of isosexual precocious puberty. Investigations revealed excessive adrenal secretion of estrogen and dehydroepiandrosterone sulfate, with normal early morning cortisol. Though overnight dexamethasone suppression test is important to exclude subclinical hypercortisolism, and pheochromocytoma screening test is mandatory, these tests were not done due to financial constraints. Not performing a workup for pheochromocytoma before adrenal mass surgery can put the patient at risk of hypertensive crisis. These are limitations of our case report.

Imaging using ultrasonography is cheap and easy, but it has low sensitivity and specificity in the characterization of adrenocortical neoplasms. It can play a pivotal role in neonates and young children as the adrenal gland has a bigger size in this age group [4]. Computed tomography (CT) is currently the first imaging modality in the evaluation and diagnosis of adrenal lesions. It helps in positioning, staging, and operative plan formulation [1]. But the imaging characteristics of different adrenal tumors are similar, and it is hard to differentiate benign from malignant tumors based on imaging alone.

To define the benign or malignant nature of the adrenal tumor is difficult, even in pathology. Three histopathological scoring systems can be used to predict the malignant nature of the adrenal tumors. Weiss score and modified Weiss score, which are used to differentiate adrenocortical adenoma (ACA) from adrenocortical carcinoma (ACC) in adults have low accuracy in the pediatric age group, so are replaced by the Wieneke index [5].

Therefore, to define malignant from the benign nature of adrenal tumor clinical behavior, laboratory, imaging, and pathological findings need to be considered.

ACAs can be successfully treated by total excision. Either laparoscopically if the tumor size is less than 10 cm, or by open surgery for larger tumors. Early detection and total excision by open surgical approach are the main steps in managing ACC as partial excision and advanced stage are associated with poor outcome. Mitotane can be used as adjuvant therapy in ACC [6]. Aromatase inhibitors are drugs that inhibit estrogen synthesis from androgens, so they can be used in FAT when there is residual estrogen secretion due to partial surgical removal, or as palliative treatment in advanced metastatic disease. Chemotherapy can be used for malignant tumors [2].

The prognosis for benign adrenocortical tumors is good, except for the risk of recurrence, while malignant adrenocortical tumors have a poor prognosis with a 5-year survival rate of 49–55% [7].

Estradiol E2 assessment using fluorometric immunoassay, which is available in our setting, can detect high estrogen secretion, the main biochemical finding in FAT. The peak incidence of adrenocortical tumors is in those younger than 5 years, so ultrasonography can be used as a screening method in our limited-resource setting for these young patients with peripheral precocious puberty. They have relatively larger adrenal glands that can be visualized using ultrasonography. So recommending estradiol level assessment and ultrasonography as part of standard assessment tools of patients with precocious puberty can aid early diagnosis and management of FATs.

## Conclusion

Adrenocortical tumors, though rare in pediatrics, are documented cause of precocious puberty, so biochemical and imaging screening protocol should be adopted even in a resource-limited setting for early detection and treatment. Assessment of estradiol level combined with abdominal ultrasound can aid in the early detection of feminizing adrenocortical tumors.

### Patient perspective

Finally, the family had found an answer to why their kid was developing breasts since the age of 9 months, why she was growing faster compared with her peers, and they were happy with their management, which helped to stop early pubertal progression.

## Data Availability

Not applicable.

## Abbreviations

ACA: Adrenocortical adenoma
ACC: Adrenocortical carcinoma
ACT: Adrenocortical tumor
CT: Computed tomography
FAT: Feminizing adrenocortical tumors
